# Bias problems in culture-independent analysis of environmental bacterial communities: a representative study on hydrocarbonoclastic bacteria

**DOI:** 10.1186/2193-1801-2-369

**Published:** 2013-08-01

**Authors:** Husain Al-Awadhi, Narjis Dashti, Majida Khanafer, Dina Al-Mailem, Nidaa Ali, Samir Radwan

**Affiliations:** Department of Biological Sciences, Faculty of Science, Kuwait University, P.O. Box 5969, Safat, 13060 Kuwait

## Abstract

Culture-dependent methods for bacterial community analysis are currently considered obsolete; therefore, molecular techniques are usually used instead. The results of the current study on hydrocarbonoclastic bacteria in various oily habitats in Kuwait showed however, that the bacterial identities varied dramatically according to the analytical approach used. For six desert and six seawater samples used in this study, the culture-independent and culture-dependent techniques each led to a unique bacterial composition. Problems related to the culture-dependent technique are well known. The results of the current study highlighted bias problems other than those already recorded in the literature for the molecular approaches. Thus, for example, in contrast to the culture-dependent technique, the primers used in the molecular approach preferentially amplified the 16S rDNAs of hydrocarbonoclastic bacteria in total genomic DNAs of all the studied environmental samples, and in addition, failed to reveal in any environmental sample members of the Actinobacteria. The primers used in the molecular approach also amplified certain “pure” 16S rDNAs, but failed to do so when these DNAs were in mixture. In view of these results, it is recommended that the two analytical approaches should be used simultaneously because their combined results would reflect the bacterial community composition more precisely than either of them can do alone.

## Introduction

The end of the 19^th^ century witnessed the development of the well known and long adopted culture-dependent approach which is used for the study of the structure of the bacterial communities in various environments. This approach drove advances in microbiology, in spite of its well known, serious limitations (Amann et al. 1995; Jannasch & Jones 1959), mainly related to the selectivity of the nutrient media and culture conditions which lead to favoring only a fraction of the inhabiting bacterial community. The major limitation of this classical technique is thus, that it dramatically underestimates the microbial numbers and composition in the samples under study. On the other hand, the major advantage of this approach over the modern molecular techniques lies in that it provides the researcher with the microbial “material” that can be used in further studies.

Within the past few decades, molecular approaches have been developed (Head et al. 1998; Jannasch & Jones 1959; Muyzer & Smalla 1998) and used as alternative methods, neglecting in most studies the culture-depedent techniques, which are considered by most modern microbiologists as “obsolete”. The molecular methods “provide a new insight into microbial diversity and allow a more rapid, high resolution description of microbial communities than that provided by the traditional approach of isolation of microorganisms” (Dahllöf et al. 2000). Molecular approaches which comprise among others the combination of DGGE fingerprinting with sequencing of 16S rDNA bands to identify the species present in the environmental samples (Fuhrman & Davies 1997; Nielsen et al. 1999; Rölleke et al. 1999), and correlating the banding patterns and band numbers on DGGE gels with environmental variables (El Fantroussi et al. 1999; Nübel et al. 1999; Sievert et al. 1999; van Hannen et al. 1999a; van Hannen et al. 1999b) also have their limitations. Serious artifacts (Polz & Cavanaugh 1998; Sipos et al. 2007) do arise viz preferential amplification of upcoming species, inadequate specificity of primers used for DNA amplification, production of single bands by multiple strains and others.

During two decades of research on microorganisms in pristine and oily Kuwaiti areas that were polluted via the greatest man-made oil-pollution catastrophy in association with the second Gulf War, 1990/1991 (Al-Awadhi et al. 2012), we consistently noticed that microbial identities determined by using culture-dependent methods for analysis were dramatically, sometimes completely different from those determined using molecular approaches. Here, we demonstrate this in a microbiological study on hydrocarbon-utilizing bacterial community structures in seawater and desert samples with over 20 years history of heavy oil-pollution. We concluded from the results that the simultaneous use of the traditional culture-dependent methods, along with the modern molecular techniques is a must for obtaining precise findings from such studies.

## Materials and methods

**Figure 1:**
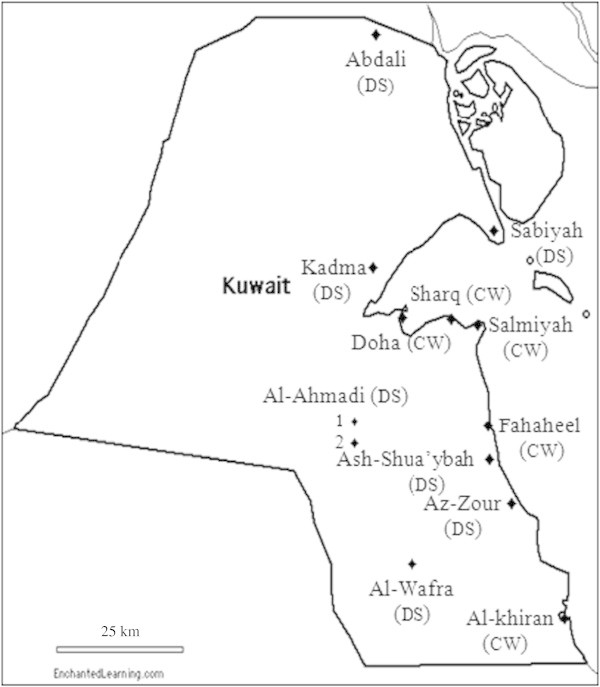
**Kuwait map showing the coastal water (CW) and desert soil (DS) sampling sites.**

Oily and pristine seawater and desert soil samples were collected from various sites (Figure 1) along the Arabian Gulf coast, and from the desert of Kuwait, and processed the same day. As the culture-dependent method, we adopted the traditional dilution-plate method using a mineral medium with oil-vapor as the sole source of carbon and energy (Al-Awadhi et al. 2012). Representative colonies of oil vapor-utilizing bacteria were isolated and purified, their total DNA’s were extracted, the 16S rRNA genes were amplified using the universal primer pair GM5F and 907R, the amplicons were sequenced and the sequences compared with the nearest GenBank sequences (method details are available in reference (Al-Awadhi et al. 2012).

For the molecular analysis of the environmental samples, the total genomic DNAs in the latter were extracted using Rapid Water DNA Isolation Kit (MO-BIO, Carlsbad, CA) for seawater samples and Fast DNA Spin for Soil Kit (MP Biomedicals, LLC., France) for desert soil samples. The extracts were stored at −80°C until used. The 16S rRNA-genes in the purified extracts were amplified as described earlier (Al-Awadhi et al. 2012). The 16S rDNA amplicons were subjected to parallel DGGE using Dcode Universal Mutation Detection System (Bio-Rad, California, USA). The denaturant concentrations were 45–55% for seawater samples and 45-60% for desert soil samples. DGGE was processed with the constant voltage of 50 V at 60°C for 16 h. Gels were stained with SYBR Green (Invitrogen, USA) in 1× TAE buffer (1:100000) for 30 min, and examined using a Dark Reader transilluminator (Clare Chemical Research, CO, USA). The bands were transformed into binary matrix; the presence of bands was given the weight of (1) and their absence (0). The binary matrix produced was analyzed using cluster analysis and dendrograms were plotted. For identification of individual bands; gel bands were excised, stored in 50 μl molecular water (Sigma, UK) at 4°C overnight to elute the DNA, 1 μl of the eluted DNA was amplified using the above primers, sequenced and the sequences were compared with the sequences in the GenBank database.

## Results

The results of the culture-dependent analysis of the oil-polluted seawater and desert soil samples for the structure of their hydrocarbon-utilizing bacterial communities have been published before (Al-Awadhi et al. 2012), and are mentioned here only for comparison with the results of the current molecular analysis. The same environmental samples were used the same sampling day for total DNA extraction; the extracts were stored at −80°C and used in the current study. Details of environmental conditions that were prevailing during sampling have been described elsewhere in details (Al-Awadhi et al. 2012).

**Figure 2:**
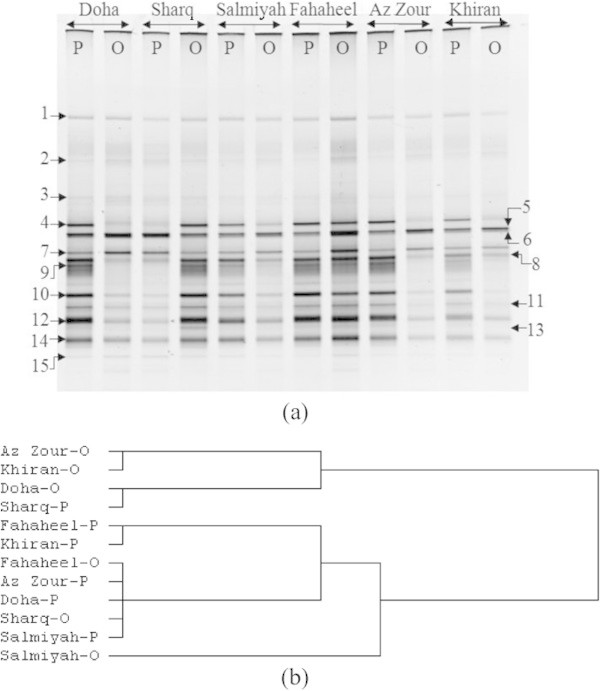
**DGGE of 16S rDNA amplicons in total DNA extracts from six pristine (P) and nearby six oil-polluted (O) seawater samples collected from the Arabian Gulf coast of Kuwait. (a)** DGGE gel; in contrast to the cases of the “closed” desert samples (Figure 3), the DGGE bands of the “open” seawater samples were similar as far as the band numbers and patterns are concerned. The total numbers of the bands for all samples were not much higher than the total number of species recorded in each sample using culture-based analysis (Al-Awadhi et al. 2012). DNAs in individual bands were amplified, sequenced and the sequences were compared with those (cultured and uncultured) of the closest species in the GenBank (results in Table 1, which presents also information related to the sequencing). **(b)** Cluster analysis of DGGE-results using Euclidean distances.

**Figure 3:**
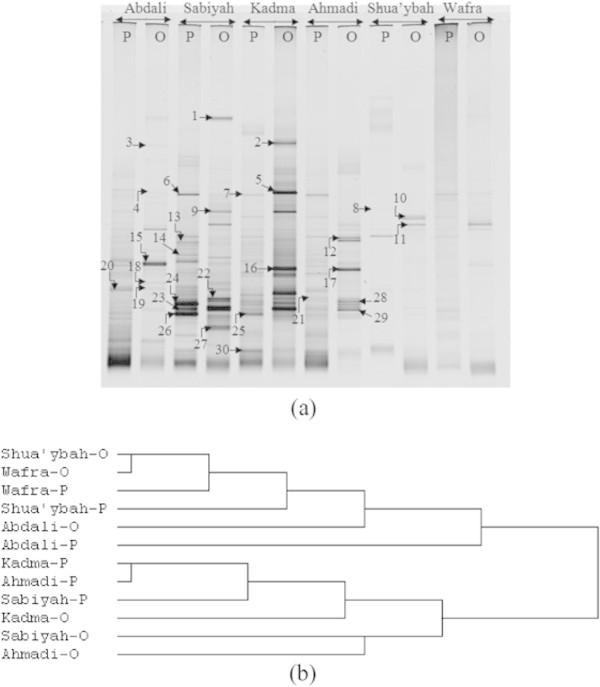
**DGGE of 16S rDNA amplicons in total DNA extracts from six pristine (P) and nearby six oil-polluted (O) soil samples collected from the Kuwaiti desert. (a)** DGGE gel, the 16S rDNA band numbers and patterns varied, not only according to the sampling sites, but also to whether the sample was pristine or oil-polluted. The bands were further processed as described in the legend to Figure 1, and the results are summarized in Table 2. **(b)** Cluster analysis of DGGE-results using Euclidean distances.

The results of the DGGE analysis of partially amplified 16S rDNA of the studied samples are presented in Figures 2 and 3. For the purpose of comparison, we also co-analyzed nearby, visually pristine samples. The DGGE profiles of the various seawater samples (Figure 2) showed a high degree of similarity as far as the 16S rDNA band numbers and migration patterns are concerned. This similarity was also valid for the profiles of the same sample irrespective of whether it was pristine or oil-polluted. In contrast, the DGGE profiles of the desert soil samples (Figure 3) exhibited dramatic variations, also depending on whether the sample was pristine or oil-polluted.

The 16S rDNA bands in Figures 2 and 3 were amplified, sequenced and the sequences were compared with the nearest sequences in the GenBank database. Like it was the case in many of our (and other) earlier reports, we failed to sequence many of the bands excised from the DGGE-gel. The results of this analysis (Tables 1 and 2) revealed only very little similarity but dramatic differences in comparison with the results previously obtained by the culture-dependent method (Al-Awadhi et al. 2012).

**Table 1:** **Results of 16S rDNA sequencing of DGGE-bands of seawater samples in Figure **2

Band no.	Total bases	Phylum	Nearest GenBank match	Similarity (%)	Bases compared	Accession number
1	446	α-Proteobacteria	*Candidatus Pelagibacter* ubique clone fosmid 01-003783	97	466/482	KC147468
2	444	Bacteroidetes	Uncultured Bacteroidetes* bacterium clone CB22B12	97	461/477	KC147469
3	455	γ-Proteobacteria	*Alteromonas macleodii* strain CAIM 891	96	494/513	KC147470
4	402	γ-Proteobacteria	*Alteromonas macleodii* strain CAIM 891	93	464/499	KC147471
5	442	γ-Proteobacteria	*Thalassolituus oleivorans* isolate SLHC162b	96	490/513	KC147472
6	450	α-Proteobacteria	*Phaeobacter caeruleus* strain UDC410	98	468/477	KC147473
7	447	α-Proteobacteria	*Phaeobacter caeruleus* strain UDC410	98	465/474	KC147474
8	455	γ-Proteobacteria	*Alteromonas macleodii* strain SWDOH3	97	488/504	KC147475
9	470	γ-Proteobacteria	*Pseudoalteromonas phenolica*	97	504/521	KC147476
10	438	α-Proteobacteria	*Thalassobius mediterraneus* strain D6064	95	466/489	KC147477
11	438	γ-Proteobacteria	*Alteromonas macleodii* strain D7081	94	487/517	KC147478
12	405	γ-Proteobacteria	*Alteromonas macleodii* strain CAIM 891	94	452/481	KC147479
13	430	γ-Proteobacteria	*Marinobacterium marisflavum* strain IMCC4074	94	482/515	KC147480
14	438	γ-Proteobacteria	*Marinobacterium marisflavum* strain IMCC4074	96	485/506	KC147481
15	440	γ-Proteobacteria	Uncultured gammaproteobacterium clone OTU29	95	477/501	KC147482

*Hydrocarbon-utilizers (Scherr et al. 2012; Jiménez et al. 2007).

**Table 2:** **Results of 16S rDNA sequencing of DGGE-bands of desert soil samples in Figure **3

Band no.	Total bases	Phylum	Nearest GenBank match	Similarity (%)	Bases compared	Accession number
1	462	Flavobacteriia	*Salinimicrobium xinjiangense* strain BH206	98	486/498	KC147483
2	493	Sphingobacteriia	*Sediminibacterium* sp. nju-T3	99	494/495	KC147484
3	423	Sphingobacteriia	*Segetibacter koreensis* strain Gsoil 664	95	476/502	KC147485
4	500	β-proteobacteria	*Ralstonia solanacearum* strain in4ss52	100	500/500	KC147486
5	512	β-proteobacteria	*Ralstonia solanacearum* strain in4ss52	100	512/512	KC147487
6	493	β-proteobacteria	*Naxibacter alkalitolerans* strain A12	99	503/510	KC147488
7	519	γ-Proteobacteria	*Acinetobacter junii* strain OVC9	100	519/519	KC147489
8	502	Synergistetes	Synergistaceae* bacterium enrichment culture clone B6_95	100	502/502	KC147490
9	510	γ-Proteobacteria	*Acinetobacter junii* strain OVC9	100	510/510	KC147491
10	469	γ-Proteobacteria	Uncultured Chromatiales† bacterium isolate DGGE gel band B16	98	491/502	KC147492
11	468	γ-Proteobacteria	Uncultured Chromatiales bacterium isolate DGGE gel band B16	98	490/501	KC147493
12	501	Synergistetes	*Synergistaceae* bacterium enrichment culture clone B6_95	99	504/505	KC147494
13	462	Cytophagia	*Pontibacter akesuensis* strain AKS 1	96	503/523	KC147495
14	447	Cytophagia	*Pontibacter xinjiangensis* strain: NBRC 107674	95	498/523	KC147496
15	376	Firmicutes	Bacillales bacterium Mi4	91	457/503	KC147497
16	421	Firmicutes	Firmicutes bacterium enrichment culture clone BSK_60	95	465/487	KC147498
17	463	β-proteobacteria	*Curvibacter delicatus* strain: NBRC 14919	97	500/518	KC147499
18	513	Firmicutes	*Planomicrobium glaciei* strain L25	99	515/516	KC147500
19	494	Firmicutes	*Planomicrobium alkanoclasticum* strain QT3+	99	498/502	KC147501
20	498	Firmicutes	*Bacillus niacini* strain GYR1	99	502/506	KC147502
21	480	β-proteobacteria	*Burkholderia oxyphila* clone: pCR2.1::OX-01_rDNA#7	97	499/512	KC147503
22	425	Firmicutes	*Bacillus selenatarsenatis* strain NBSL41	96	458/475	KC147504
23	501	Firmicutes	*Planomicrobium glaciei* strain GDM825	99	502/503	KC147505
24	493	Firmicutes	*Planomicrobium okeanokoites*	99	494/495	KC147506
25	475	Firmicutes	*Planomicrobium okeanokoites* strain QL-25	98	486/494	KC147507
26	514	Firmicutes	*Planomicrobium glaciei* strain L25	99	516/517	KC147508
27	427	Firmicutes	Uncultured *Geobacillus* sp. clone SHBZ1548	94	484/513	KC147509
28	464	Firmicutes	*Bacillus selenatarsenatis* strain NBSL41	95	496/521	KC147510
29	505	Firmicutes	*Bacillus selenatarsenatis* strain NBSL41	99	506/507	KC147511
30	475	γ-Proteobacteria	*Halomonas xinjiangensis* strain YIM 91125	97	502/518	KC147512

*Hydrocarbon-utilizers (Scherr et al. 2012).

† Hydrocarbon-utilizers (Jiménez et al. 2007).

The slight similarity was expressed in the predominance of the phylum Gammaproteobacteria in seawater and its relative rarity in the terrestrial samples. Assuming that each DGGE band on the gel represents one single species, as commonly accepted, our results demonstrate that the species numbers determined by using the molecular analysis for all the environmental samples were more than the numbers determined by using the culture-dependent method, and the differences were more pronounced for the desert than the seawater samples (Table 3). For the seawater samples, the differences ranged between about 1 and 4 fold, whereas for the desert samples, they ranged between about 1 and 12 fold more species counted by the molecular than by the culture-dependent methods.

**Table 3:** **Comparison between the composition of hydrocarbon-utilizing bacterial communities in seawater and desert soil-samples determined by the culture-based method versus that determined by a modern molecular approach**

Sampling sites	Culture-based analysis (using a mineral medium with oil vapor as sole source of carbon and energy, detailed results in Al-Awadhi et al. 2012)			Combined DGGE and band amplification analysis
	Total number of species	Affiliated to the hydrocarbon-utilizing genera:	Total number of DGGE bands	Affiliated to the genera (hydrocarbon-utilizers are designated with the pertinent reference numbers):
Seawater				
Doha	17	*Psychrobacter, Oceanobacillus, Vibrio, Agarivorans,****Alteromonas****, Marinobacter, Stappia,****Pseudoalteromonas****, Microbacterium, Marinomonas, Nesiotobacter, Mycobacterium*	18	***Alteromonas****, Candidatus*^1^*, Marinobacterium, Phaeobacter, Thalassobius*^2^*, Thalassolituus*^3^
Sharq	5	*Alcanivorax, Stappia, Thalassospira, Nitratireductor*	20	***Alteromonas****, Candidatus, Marinobacterium, Phaeobacter,****Pseudoalteromonas****, Thalassobius, Thalassolituus*
Salmiyah	15	***Alteromonas****, Echinicola, Klebsiella, Alcanivorax, Marinomonas, Gordonia, Pseudomonas, Rhodococcus, Microbacterium, Vibrio, Marinobacter, Kocuria*	17	***Alteromonas****, Candidatus, Marinobacterium, Phaeobacter,****Pseudoalteromonas****, Thalassobius, Thalassolituus*
Fahaheel	9	*Pseudomonas, Dietzia, Shewanella, Arthrobacter,****Pseudoalteromonas****, Acinetobacter,****Alteromonas***	20	***Alteromonas****, Candidatus, Marinobacterium, Phaeobacter,****Pseudoalteromonas****, Thalassobius, Thalassolituus*
Az Zour	4	*Alcanivorax, Cobetia,****Pseudoalteromonas***	15	***Alteromonas****, Candidatus, Marinobacterium, Phaeobacter, Thalassobius, Thalassolituus*
Al Khiran	6	*Alcanivorax,****Alteromonas****,****Pseudoalteromonas****, Cobetia*	15	***Alteromonas***, Candidatus, Marinobacterium, Phaeobacter*,***Pseudoalteromonas****, Thalassobius, Thalassolituus*
Desert soil				
Al Abdali	7	*Arthrobacter, Dietzia, Microbacterium, Streptomyces, Agrococcus*	30	*Bacillus, Planomicrobium*^4^*, Ralstonia*^5^*, Salinimicrobium*^6^*, Segetibacter*^7^
Sabiyah	8	*Microbacterium, Dietzia, Pseudomonas, Bordetella, Roseomonas,*	36	*Acinetobacter, Bacillus, Burkholderia*^8^*, Geobacillus*^9^*, Halomonas*^10^*, Naxibacter*^11^*, Planomicrobium, Ralstonia*
Kadma	4	*Pseudomonas, Sphingomonas*	47	*Acinetobacter, Bacillus, Burkholderia, Curvibacter*^5^, Sediminibacterium^*^, Halomonas, Naxibacter, Planomicrobium, Pontibacter^12^*, Ralstonia, Segetibacter*
Ahmadi	6	*Cellulomonas, Pseudomonas, Arthrobacter, Sphingomonas*	30	*Acinetobacter, Bacillus, Burkholderia, Curvibacter, Naxibacter, Planomicrobium, Pontibacteter*
Ash Shua’yba	12	*Mycobacterium, Nocardia, Rhodococcus, Streptomyces,****Bacillus***	17	*Bacillus, Planomicrobium, Pontibacter*
Al Wafra	8	*Kocuria, Streptomyces, Agrobacterium,****Acinetobacter****, Pseudomonas, Brevundimonas, Sphingobium*	17	***Acinetobacter****,****Bacillus****, Halomonas, Naxibacter*

*No references were found on the hydrocarbon-degradation potential of *Sediminibacterium* or *Phaeobacter*. The superscript numbers are the reference numbers in the list recording hydrocarbonoclastic activity among species belonging to the given genera (1, Prabagaran et al. 2007;2, Teramoto et al. Teramoto et al. 2009; 3, Yakimov et al. Yakimov et al. 2004; 4, Yakimov et al. Yakimov et al. 2007; 5, Zhu et al. Zhu et al. 2010; 6, Yergeau et al. Yergeau et al. 2012; 7, Larentis et al. Larentis et al. 2009; 8; Morawski et al. 1997; 9, Arun et al. Arun et al. 2011; 10, Wang et al. Wang et al. 2007; 11, Kleinsteuber et al. Kleinsteuber et al. 2006; 12, Wan et al. Wan et al. 2011).

Genera that were revealed by both approaches are in bold.

**Figure 4:**
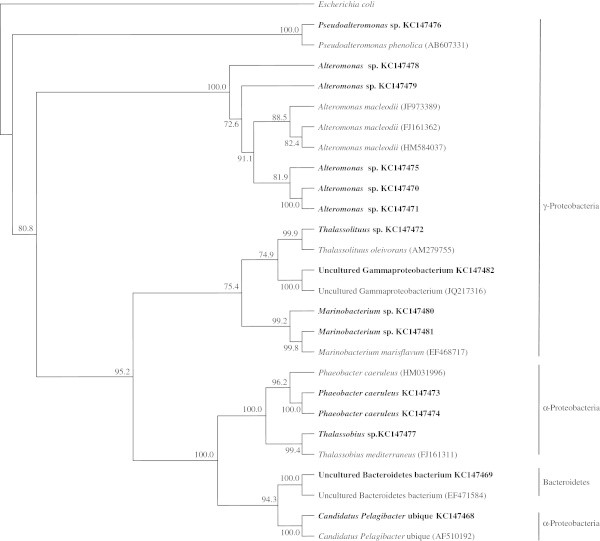
**Phylogenatic tree of 16S rRNA genes of bacteria from Kuwaiti oil polluted coastal water, as analyzed by the culture-independent method.** Values shown in each node of the phylogenetic tree are bootstrap value; 2,000 bootstrap replicates were performed.

**Figure 5:**
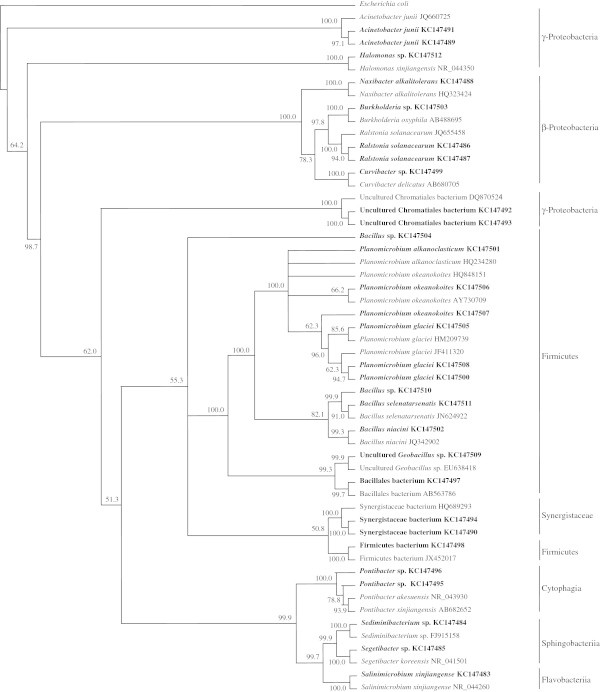
**Phylogenatic tree of 16S rRNA genes of bacteria from Kuwaiti oil polluted desert soil, as analyzed by the culture-independent method.** Values shown in each node of the phylogenetic tree are bootstrap value; 2,000 bootstrap replicates were performed.

In none of the six desert samples investigated could any single species be simultaneously recorded in the composition lists obtained by using the two techniques. Even the species belonging to the genera *Bacillus* and *Acinetobacter* in Ash Shua’yba and Al-Wafra samples, respectively varied according to the analytical approach. Using the culture-dependent method (Al-Awadhi et al. 2012), we recorded *B*. *infantis* and *A*. *septicus*, whereas by the molecular analysis, we recorded *B*. *niacini*, *B*. *selenatarsenatis* and *A*. *junii*. Basically, the same observation, albeit at a slightly less pronounced level, could be made for the open seawater samples. In two samples; Sharq and Az Zour the bacterial species lists were totally different depending on the analytical technique. In the other remaining seawater samples, the molecular approach revealed only two species, *Alteromonas macleodii* and *Pseudoalteromonas phenolica*, which were also recorded by the culture-dependent method (Al-Awadhi et al. 2012). The remaining species were totally different, as obvious in Table 3. The analytical technique-dependent differences of the bacterial community composition are also quite obvious in the phylogenetic trees based on the bacteria analyzed by the molecular approach in Figures 4 & 5 as compared with the corresponding trees based on bacteria analyzed by the traditional culture-deoendent method (Al-Awadhi et al. 2012).

For each of the 24 studied environmental samples, the bacterial community composition determined by using the culture-dependent method (Al-Awadhi et al. 2012) was dramatically, sometimes totally different from that determined by using the molecular approach. This implies that none of the two techniques can be used as a substitute of the other, since each of them favors bacterial taxa different from these favored by the other. Consequently, in order to limit the bias, both approaches should be adopted simultaneously, and the collective result would obviously reflect the “real bacterial composition” more precisely than that obtained using either of them alone, even though the bias problems are still not rased.

To provide a further evidence for the validity of the latter conclusion, the contribution of both techniques to the analysis of the group of the “obligate oil-degrading marine bacteria” in the seawater samples is highlighted. This is a new and ecophysiologically unusual group recently recognized and termed “the obligate hydrocarbonoclastic bacteria (OHCB)”; they repotedly play the major role in bioremediating oil in the marine ecosystem (Yakimov et al. 2007). The most common OHCB are species belonging to a few genera; *Alcanivorax*, *Marinobacter*, *Thalassolituus*, *Cycloclasticus* and *Oleispira* that occur in pristine seawater in minute numbers, but bloom immediately after oil-pollution. Our results show that *Alcanivorax* described as the “paradigm” of the OHCB (Yakimov et al. 2007), and *Marinobacter* were recorded, respectively, in 4 and 2 of the studied seawater sites, but only by using the culture-dependent analytical method (Al-Awadhi et al. 2012). The molecular approach we adopted failed to reveal any of the two OHCB genera in any of the six seawater samples. On the other hand, the genus *Thalassolituus* was recorded in all six sampling sites, but only when the molecular approach was adopted. By neither of the two techniques could *Cycloclasticus* and *Oleispira* be recorded in any of the six seawater samples. Probably, the two genera were not present in the studied samples. Interestingly however, using the molecular (but not the culture-dependent) approach, we recorded the former genus in biofilms on glass plates submerged in pristine and oily Gulf-water (Al-Bader et al. 2012).

## Discussion

The DGGE profile similarities of the different seawater samples (Figure 1) should be expected, in view of that seawater in situ is an “open” environment in which the whole water body becomes mechanically mixed up to some extent. Remote samples would thus, show a degree of similarity in their microbial composition. Meanwhile, the DGGE dissimilarities of the different desert soil samples (Figure 2) may be explained on the basis that the processes regulating the bacterial species frequencies in various localities, the so-called patchiness operate at a very narrow, sometimes centimeter spacial scale (Duarte & Vaqué 1992; Long & Farooq 2001; Seuront et al. 2002; Seymour et al. 2000). Expectedly, this phenomenon should be more pronounced in the “closed”, terrestrial than the “open”, aquatic environments. The observed frequent occurrence of the Gammaproteobacteria in the Arabian Gulf water body has also often been recorded by earlier investigators (Al-Awadhi et al. 2012; Al-Sarawi et al. 2008) in the marine habitats.

An important and well known advantage of the culture-dependent approach is that it routinely analyses specific groups of bacteria (in this study oil-utilizers) via the use of selective nutrient media. Since this is not the case with the molecular analysis, we had to compare our results with literature reports. The comparison confirmed that all the bacterial genera listed in Table 3, with the only exception of *Sediminibacterium* and *Phaeobacter*, comprised hydrocarbon-utilizing species (see pertinent reference numbers in Table 3). This result demonstrates a serious bias, namely that the used molecular approach preferentially amplified the 16S rDNAs of hydrocarbon-utilizing bacteria in the total DNA extracts from the environmental samples and might have “neglected” others. Still another even more pronounced bias is that the molecular approach used did not reveal in any of the studied environmental samples any member of the phylum Actinobacteria. This was true although by using the culture-dependent method, many species belonging to this phylum were found (sometimes predominant) especially in the desert soil samples (Al-Awadhi et al. 2012). Obviously the technique successfully amplifies certain 16S rDNA’s only when in pure form, but not when mixed with others, as it is the case in the environmental samples. The molecular analysis of bacterial communities on biofilms that developed on glass plates submerged in pristine and oily seawater from the Arabian Gulf (samples similar to those used in this study) also did not reveal one single species belonging to the Actinobacteria (Al-Bader et al. 2012). The present state of knowledge about this subject in the literature is too limited to explain the reason for this latter bias of preferential amplification.

It should be expected that the molecular technique would reveal in every environmental sample most, if not all of the bacterial species that showed up in these samples using the culture-dependent method. Therefore, the most surprising result in our study was that the identities of the bacteria analyzed by both techniques for all the studied samples (and for samples analyzed earlier in our laboratory) were dramatically different. In this context, the assumption that each DGGE band represents one single species is not correct (Sekiguchi et al. 2001). It is established today that differential/preferential amplification of 16S rRNA-genes in environmental samples usually leads to serious bias regarding the actual composition of the bacterial communities (Polz & Cavanaugh 1998; Sipos et al. 2007). Again the results of the current study demonstrate that the technique-dependent differences in community composition are so dramatic that both techniques should necessarily complete, and not just substitute one another in every study. This is true although both approaches are associated with own bias problems. Based on our results, the molecular approach does not seem to reveal numerically much more bacterial species (even though it reveals different rather than the same species) in the environmental samples than the culture-dependent approach, as commonly believed, so far. As shown above, the differences were in all the studied samples only between about 1 and 12 fold, in favor of the molecular approach. This may appear to contradict the well known fact that direct microscopic counts give frequently up to 10^4^ fold more bacterial cell numbers than those counted by the culture-dependent method. This implies that the cells counted microscopically are most probably members of “strains” or “varieties” belonging to a limited number of species, rather than to different species as frequently misunderstood.

As already mentioned, modern microbiologists tend to consider the traditional culture-dependent methods obsolete and prefer the use of the modern molecular approaches instead. However, this study surprisingly showed that the two approaches revealed distinctly different bacterial communities for each studied sample. This unexpected result consolidates that both techniques must not substitute one another, but should mutually complement each other, in order to gain a bacterial composition most close to reality.
